# Complete Genome Sequences of Two Strains of Streptococcus pyogenes Belonging to an Emergent Clade of the Genotype *emm*89 in Brittany, France

**DOI:** 10.1128/MRA.00129-20

**Published:** 2020-03-12

**Authors:** Marie Devaere, Sarrah Boukthir, Séverine Moullec, Emeline Roux, Dominique Lavenier, Ahmad Faili, Samer Kayal

**Affiliations:** a CHU Rennes—Service de Bactériologie et Hygiène Hospitalière, Rennes, France; b INSERM CIC-1414, Rennes, France; c INRIA/IRISA, GenScale Bioinformatics Team, Rennes, France; University of Rochester School of Medicine and Dentistry

## Abstract

The frequency of infections due to Streptococcus pyogenes M/*emm*89 strains is increasing, presumably due to the emergence of a genetically distinct clone. We sequenced two *emm*89 strains isolated in Brittany, France, in 2009 and 2010 from invasive and noninvasive infections, respectively. Both strains belong to a newly emerged *emm*89 clade 3 clone.

## ANNOUNCEMENT

Group A beta-hemolytic Streptococcus pyogenes (GAS) is a Gram-positive human pathogen associated with a variety of human diseases, with an estimated 517,000 deaths per year (1). For epidemiological surveying of GAS infections, the *emm* genotype is a worldwide accepted marker (2, 3). Within the genotype *emm*89, genome remodeling led to the emergence of a new clade variant designated clade 3, recognized to be frequently associated with invasive infections in Europe (4, 5). Whereas at the beginning of our epidemiological survey (2009), the proportion of *emm*89 among all *emm* genotypes was less than 10%, it reached 29% in 2017 and represents the most dominant genotype in Brittany, France.

In order to decipher whether the genotype *emm*89 belonging to clade 3 was already circulating in French Brittany as early as 2009, we sequenced the whole genomes of two strains isolated in the university hospital of Rennes from a case of noninvasive vaginitis (2009; 39.9-year-old female) and one of invasive facial dermo-hypodermitis (2010; 2.6-year-old male), hereafter referred to as strains STAB09023 and STAB10048, respectively.

Strains were grown in Todd-Hewitt medium supplemented with 0.2% yeast (THY), and DNA was extracted as previously described (6). Libraries were prepared with the Nextera XT kit (Illumina, Inc., San Diego, CA) and subjected to paired-end sequencing (2 × 125 bp) on a HiSeq 2500 system (Illumina). The total read counts were 12,473,106 (STAB0902) and 13,677,462 (STAB10048), giving an average of 817-fold coverage for both strains. The quality of reads was assessed by FastQC (7).

Reads were assembled using CLC Genomics Workbench v. 6 software (Qiagen Bioinformatics, Aarhus, Denmark). Default parameters were used for all software. The resulting assembly consisted of 21 (STAB0902; *N*_50_, 155,832 bp) and 22 (STAB1004; *N*_50_, 162,752 bp) contigs, which were placed and oriented manually based on the *emm*89 reference genome MGAS27061 (8). All the persistent gaps were filled by PCR, followed by Sanger sequencing of the amplified sequences, and the gapless genomes consisted of 1,741,934 bp (STAB0902) and 1,742,165 bp (STAB10048), with a G+C content of 38.5% for each of them. Annotations were performed by using the RAST server (9) and NCBI PGAP (http://ncbi.nlm.nih.gov/genome/annotation_prok), and prophages were examined using the PHAge search tool (PHAST) (10). For both genomes, we identified a total of 17,383 coding sequences (CDSs), 15 rRNAs, 57 tRNAs, 2 complete CRISPR sequences, and 2 integrated prophages that vary in G+C content from 37.6 to 38.7%. In particular, we found a prophage element that carried the superantigen SpeC (*speC*) and the DNase (*spd1*) genes that, as previously described, were not found in strains outside the emergent clade 3 (4). The difference of 231 bp between our two strains is mainly located in noncoding regions and the polymorphic *sclB* gene. By comparison with the MGAS27061 genome, we identified in both strains the 27 single nucleotide polymorphisms (SNPs) described as shared by all the acapsular clade 3-associated strains of the genotype *emm*89 (4).

Since 2009, the *emm*89 genotype belonging to an emergent clade has been circulating in French Brittany. Herein, we have made available 2 complete sequences that could facilitate evolutionary studies of this highly invasive clone, which could provoke intrahospital epidemics and rapidly spread in the world (11).

### Data availability.

The complete genome sequences of these S. pyogenes strains have been deposited in NCBI GenBank and the raw data into the NCBI Sequence Read Archive (SRA). The genome versions described in this paper are the first versions. The BioProject, GenBank, and SRA accession numbers are PRJNA524538, CP036530, and SRP237255 (PRJNA524538) for STAB09023 and PRJNA524780, CP036531, and SRP237258 (PRJNA524780) for STAB10048, respectively.
